# Pre-stroke cognitive impairment is associated with vascular imaging pathology: a prospective observational study

**DOI:** 10.1186/s12877-021-02327-2

**Published:** 2021-06-14

**Authors:** Till Schellhorn, Manuela Zucknick, Torunn Askim, Ragnhild Munthe-Kaas, Hege Ihle-Hansen, Yngve M. Seljeseth, Anne-Brita Knapskog, Halvor Næss, Hanne Ellekjær, Pernille Thingstad, Torgeir Bruun Wyller, Ingvild Saltvedt, Mona K. Beyer

**Affiliations:** 1 Institute of Clinical Medicine, University of Oslo, Oslo, Norway; 2 Division of Radiology and Nuclear Medicine, Oslo University Hospital, Oslo, Norway; 3 Oslo Centre for Biostatistics and Epidemiology, Department of Biostatistics, Faculty of Medicine, University of Oslo, Oslo, Norway; 4 Department of Neuromedicine and Movement Science, Faculty of Medicine and Health Science, NTNU-Norwegian University of Science and Technology, Trondheim, Norway; 5 Department of Medicine, Vestre Viken Hospital Trust, Bærum Hospital, Drammen, Norway; 6 Department of Neurology, Oslo University Hospital, Oslo, Norway; 7 Medical Department, Ålesund Hospital, Møre and Romsdal Health Trust, Ålesund, Norway; 8 Department of Geriatric Medicine, Oslo University Hospital, Oslo, Norway; 9 Department of Neurology, Haukeland University Hospital, Bergen, Norway; 10 Institute of Clinical Medicine, University of Bergen, Bergen, Norway; 11 Stroke Unit, Department of Internal Medicine, St. Olavs Hospital, Trondheim University Hospital, Trondheim, Norway; 12 Department of Geriatric Medicine, Department of Internal Medicine St. Olavs Hospital, Trondheim University Hospital, Trondheim, Norway

**Keywords:** Stroke imaging, Cognitive impairment, Pre-stroke cognitive impairment, White matter lesions, Medial temporal lobe atrophy, Sex differences

## Abstract

**Background:**

Chronic brain pathology and pre-stroke cognitive impairment (PCI) is predictive of post-stroke dementia. The aim of the current study was to measure pre-stroke neurodegenerative and vascular disease burden found on brain MRI and to assess the association between pre-stroke imaging pathology and PCI, whilst also looking for potential sex differences.

**Methods:**

This prospective brain MRI cohort is part of the multicentre Norwegian cognitive impairment after stroke (Nor-COAST) study. Patients hospitalized with acute ischemic or hemorrhagic stroke were included from five participating stroke units. Visual rating scales were used to categorize baseline MRIs (*N* = 410) as vascular, neurodegenerative, mixed, or normal, based on the presence of pathological imaging findings. Pre-stroke cognition was assessed by interviews of patients or caregivers using the Global Deterioration Scale (GDS). Stroke severity was assessed with the National Institute of Health Stroke Scale (NIHSS). Univariate and multiple logistic regression analyses were performed to investigate the association between imaging markers, PCI, and sex.

**Results:**

Patients’ (*N* = 410) mean (SD) age was 73.6 (±11) years; 182 (44%) participants were female, the mean (SD) NIHSS at admittance was 4.1 (±5). In 68% of the participants, at least one pathological imaging marker was found. Medial temporal lobe atrophy (MTA) was present in 30% of patients, white matter hyperintensities (WMH) in 38% of patients and lacunes in 35% of patients. PCI was found in 30% of the patients. PCI was associated with cerebrovascular pathology (OR 2.5; CI = 1.4 to 4.5, *p* = 0.001) and mixed pathology (OR 3.4; CI = 1.9 to 6.1, *p* = 0.001) but was not associated with neurodegeneration (OR 1.0; CI = 0.5 to 2.2; *p* = 0.973). Pathological MRI markers, including MTA and lacunes, were more prevalent among men, as was a history of clinical stroke prior to the index stroke. The OR of PCI for women was not significantly increased (OR 1.2; CI = 0.8 to 1.9; *p* = 0.3).

**Conclusions:**

Pre-stroke chronic brain pathology is common in stroke patients, with a higher prevalence in men. Vascular pathology and mixed pathology are associated with PCI. There were no significant sex differences for the risk of PCI.

**Trial registration:**

NCT02650531, date of registration: 08.01.2016.

**Supplementary Information:**

The online version contains supplementary material available at 10.1186/s12877-021-02327-2.

## Background

Stroke is a leading cause of death with high morbidity rates seen worldwide. Up to 50% of stroke survivors are left chronically disabled [1], thus posing a great burden on public health. Around 20% of stroke survivors are at risk of developing post-stroke dementia [2, 3]. To understand the impact of stroke on the risk of post-stroke dementia, pre-stroke cognitive status, including pre-stroke dementia and pre-stroke cognitive impairment (PCI), must be taken into account [4, 5]. Few studies have looked at risk factors for PCI [6]. Pre-stroke dementia is more widely studied than pre-stroke cognitive impairment and is found in around 9–14% of stroke patients [7]. Pre-stroke dementia is associated with older age at onset, greater prevalence of atrial fibrillation, a history of stroke, heart failure and premorbid use of anticoagulants and anti-hypertensive medication [8]. Additionally, female stroke survivors are more likely to suffer from pre-stroke dementia [3], which has been attributed to the fact that women generally are older than men at the time of their first stroke [9]. Pre-stroke chronic brain changes have been shown to reduce the threshold for developing post-stroke dementia [7]; moreover, changes such as cerebral atrophy [4] medial temporal lobe atrophy [9] and the number of old infarcts on CT scans [10] have been shown to be associated with PCI. In a review, Mok et al. concluded that pre-stroke Alzheimer’s disease pathology and sporadic small vessel disease are the most important pathologies associated with pre-stroke dementia [11].

Both population based studies and studies of stroke patients have measured PCI, many of them focusing on pre-stroke dementia [5, 12]. However, little is known about which neuroimaging biomarkers have the strongest association with PCI. Neurodegeneration in the form of medial temporal lobe atrophy (MTA) might occur in normal aging or as part of neurodegenerative diseases [13]. Further, neurodegeneration often coexists with cerebrovascular disease [14], but knowledge on the combined role of small vessel disease and neurodegeneration in the pathogenesis of PCI is limited.

While there is increasing interest in sex differences in ischemic and hemorrhagic stroke, it focuses mainly on epidemiology, risk factors, prevention and treatment recovery. Stroke incidence is higher in men, but more women are affected because of their longevity [15, 16]. Research on sex differences in neuroimaging markers predominantly addresses the two main imaging markers of cerebrovascular disease and neurodegeneration: white matter hyperintensities (WMH) and medial temporal lobe atrophy (MTA). WMH in particular is more severe in women [17, 18].

There is increasing evidence that female sex is a significant predictor of pre-stroke disability [19, 20], and that pre-stroke cognitive impairment leads to worse stroke outcome [21]. However, the association of neuroimaging pathology, female sex and PCI is understudied [22].

More knowledge on the extent of pre-stroke chronic pathology and its impact on PCI will help increase the understanding of the pathogenesis of post-stroke cognitive impairment. The use of readily available visual scales to evaluate the burden of both neurodegenerative and vascular brain changes gives clinicians access to important bedside information. The assessment of pre-stroke imaging pathology might help to predict the development of cognitive decline after an acute stroke.

The objectives of the current study were to 1) measure pre-stroke neurodegenerative and vascular disease pathology found on brain MRI, 2) describe the association between pre-stroke neuroimaging features and PCI, and 3) investigate possible sex differences in the risk of PCI. We hypothesized that pathological changes associated with both small vessel disease and neurodegeneration are associated with PCI and that there is a higher burden of brain changes in women.

## Materials and methods

The Norwegian cognitive impairment after stroke (Nor-COAST) study is a prospective longitudinal multi-centre cohort study [14]. Patients admitted to the stroke units at five Norwegian hospitals were recruited starting in May 2015 and ending in March 2017. Patients eligible for participation in Nor-COAST were included according to the following criteria: 1) hospitalized with acute ischemic or hemorrhagic stroke, 2) within 1 week after symptom onset, 3) age over 18 years, and 4) fluent in a Scandinavian language. Exclusion criteria were 1) symptoms explained by other disorders than ischemic brain infarct or intracerebral hemorrhages and 2) expected survival of less than 3 months after stroke based on a clinical assessment by experienced stroke physicians [14]. Inclusion criteria for MRI were 1) inclusion in Nor-COAST and 2) ability to cooperate during MRI. Exclusion criteria for MRI were 1) severe functional impairment making MRI impossible to perform; 2) medical contra-indications for MRI, such as claustrophobia or pacemaker; and 3) refusal of patient to participate in MRI.

Patients were asked to participate in the MR part of the study after they were included in the Nor-COAST study. The participation in this imaging sub-study of Nor-COAST was voluntary. As length of stay is often very short, some patients were discharged before they were asked, others lived too far from the hospital to come back after discharge. The specific reasons for the individual patient withdrawal/non-inclusion were unfortunately not recorded.

To evaluate whether the included patients are representative of the whole Nor-COAST study population, we compared the clinical characteristics and risk factors of patients with and without MRI.

### MRI acquisition

**Fig. 1:**
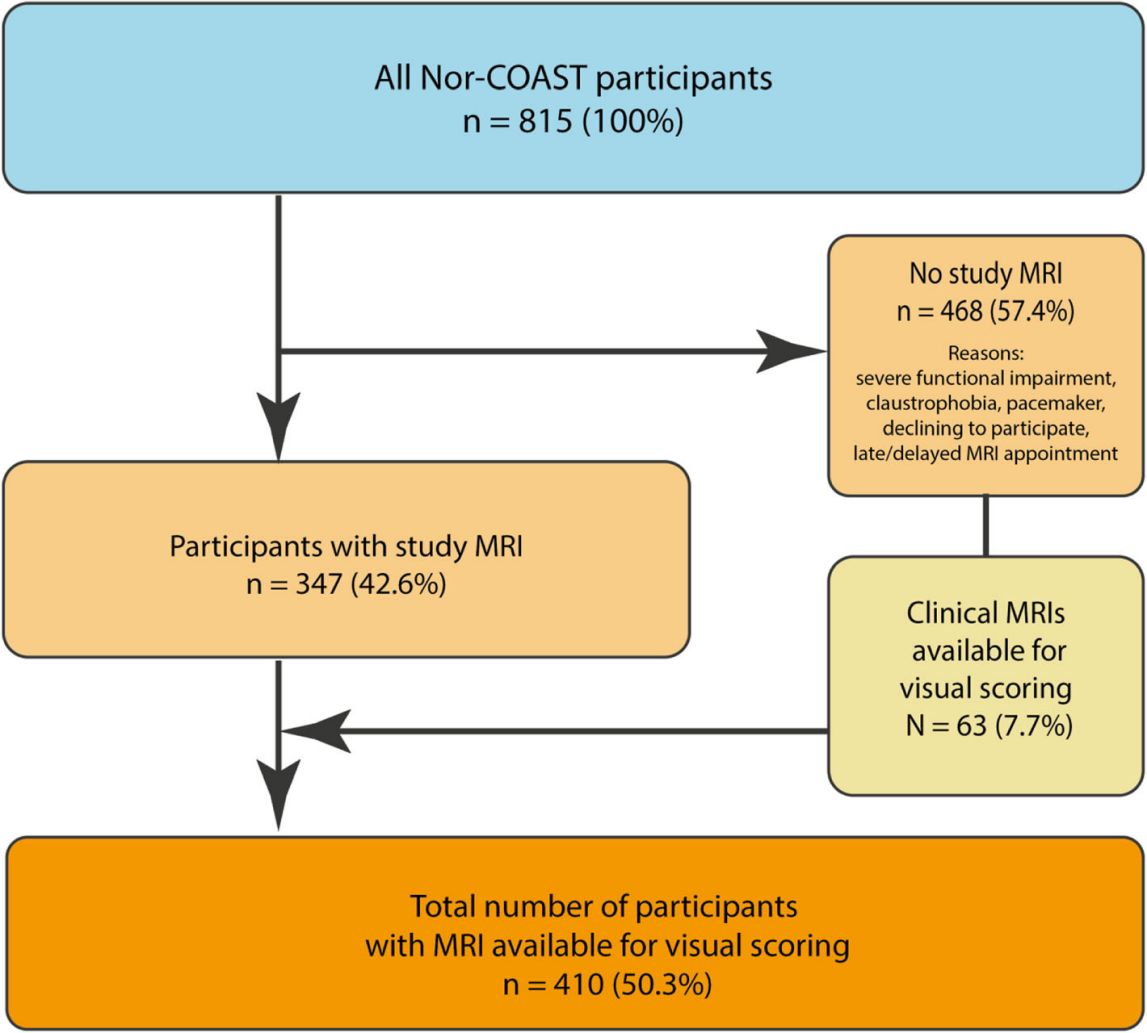
Flowchart of patient inclusion to this study

A study-specific brain MRI was acquired at one of five different sites in the acute/subacute phase of the stroke (i.e. 2–7 days after symptom onset). The study protocol consisted of a 3D T1-weighted sequence, axial T2, 3D Fluid attenuated inversion recovery (FLAIR), diffusion weighted imaging (DWI), and susceptibility weighted imaging (SWI). Details about the MRI protocol can be found in Supplementary Table 1. For a number of patients without a study-specific brain MRI, a clinical MRI scan was available for visual analysis. The clinical MRI sequences were acquired with different sequence parameters than the study-specific MRIs but were suitable for visual rating (Fig. 1).

### Image analysis

Only brain changes unrelated to acute stroke were analyzed using validated visual rating scales for markers of neurodegenerative disease [23]. Small vessel disease features were rated according to the Standards for Reporting Vascular Changes on Neuroimaging (STRIVE) recommendations [14]. The extent of white matter hyperintensities (WMH) of presumed vascular origin was classified according to the widely used Fazekas scale [24] using the 3D FLAIR sequence and categorized as normal or pathological based on the score and age [25]. Edema associated with the acute stroke lesion was not scored as WMH. Lacunes of presumed vascular origin were assessed on the 3D FLAIR sequence and categorized as present/not present; they were always regarded as pathological [26]. Parenchymal defects with significant loss of volume without corresponding diffusion restriction were considered old infarcts. Silent infarct was defined in patients with visible MRI changes in either lacunes or cortical/subcortical infarct but no record of previous clinical infarct. Microbleeds, i.e. the existence of more than or equal to two (≥ 2) hypointense lesions on SWI [27], were registered as present/not present [28]. Since microbleeds and lacunes were always regarded as pathological, their occurrence was not further evaluated. Medial temporal lobe atrophy (MTA) was assessed according to the established MTA scale [29]. MTA was categorized as normal or pathological depending on score and age [23]. Posterior atrophy was assessed according to the posterior atrophy (PA) scale [30]. A value of ≥2 was considered pathological in patients below 95 years of age [23]. Ventricular enlargement, which is an indirect measure of global cerebral atrophy, was measured using the Evans index (EI) [31] and categorized as normal or pathological using sex- and age-dependent reference values [32].

Patients were clustered into 1) a neurodegeneration group, with pathological scores for MTA, PA or EI and no pathological imaging markers of cerebrovascular disease; 2) a cerebrovascular group, with pathological scores for WMH, lacunes or microbleeds, and no pathological imaging markers of neurodegeneration; 3) a mixed group, with patients with pathological scores for imaging markers from both categories 1 and 2; and 4) a normal group, with no pathological score on visual rating of brain MRI.

### Reliability

To facilitate rating consistency between the two participating neuroradiologists (TS, MKB), a pilot dataset of 20 MRI scans was randomly selected and all relevant scales (MTA, Fazekas, PA, EI, microbleeds) were scored. The possible causes of differing results were then evaluated and discrepencies were resolved in a consensus meeting. Subsequently, 30 new randomly selected scans were scored to evaluate the inter-rater variability. The same dataset was scored again by one neuroradiologist (TS) to evaluate the intra-rater reliability (please see Supplementary figure 1 for an overview of the process). The final visual assessment of the total 410 patients included in this study was done by one neuroradiologist (TS). No cognitive assessment results were accessible by the neuroradiologists before completion of the visual ratings.

The inter-rater and intra-rater agreement measured in percent was excellent for WMH, MTA, PA, and microbleeds, and good for lacunes. The detailed results of reliability testing of visual ratings are presented in Supplementary Table 3.

### Cognitive assessment and cerebrovascular risk factors

The Global Deterioration Scale (GDS) was used for the evaluation of PCI. The GDS is a global measure of cognitive function originally designed for the evaluation of AD, but it has also been shown to be valid for measuring vascular dementia [33–36]. The assessment was performed at baseline by experienced research nurses who interviewed caregivers about the patients’ cognition the last weeks before suffering an acute stroke. The GDS is a 7-point scale with higher scores indicating more impaired cognition [36]. .Patients with a GDS score of 2 were categorized as having very mild cognitive/subjective cognitive impairment [36]. GDS of 2 or higher is the first stage on the scale that represents cognitive symptoms, and it was rated as pathological [37].

The pre-stroke modified Rankin Scale (mRS) was used to measure general functioning and the degree of disability in daily activities [38]. Criteria used in defining clinical diagnoses and risk factors in the Nor-COAST study were atrial fibrillation (AF), defined as presence of atrial fibrillation on ECG in the past or during the present hospital stay; hypertension (HT), defined by use of anti-hypertensive drugs or lifestyle modification treatment (including physical activity, weight control, heart healthy diet, and smoking cessation) at the time of the index stroke; hypercholesterolemia, defined as total cholesterol ≥6.2 mmol/L or LDL ≥ 4.1 mmol/L or using cholesterol-lowering medication; diabetes mellitus (DM), defined by presence in medical records or use of antidiabetic medication, or as HbA1c ≥ 6.5%; previous stroke, defined by a history of stroke in medical records; and stroke severity, assessed by the National Institute of Health Stroke Scale (NIHSS) [39] at admittance. The ischemic stroke subtype was defined according to the Trial of ORG 10172 in Acute Stroke Treatment (TOAST) classification [40].

### Statistical analysis

For group comparisons, the Mann-Whitney U test was used for the continuous variables, and the chi-square test was used for the binary variables. The odds ratio (OR) of having PCI was calculated through a univariate logistic regression analysis including sex, pathological imaging group, silent infarcts, hypertension, atrial fibrillation, and clinically acknowledged previous stroke. The relationship between sex and pathological imaging findings was modelled using logistic regression, with male sex as the reference group. Since age represents an important potential confounding variable, it was adjusted for in the logistic regression models. This is also true for models where brain imaging markers, which were already age-adjusted, were used as explanatory variables. The relationship between the imaging groups and the existence of PCI was modelled using a multiple logistic regression analysis with the normal group as the reference group. We additionally evaluated the effects of imaging group, age, and education as confounding variables, as these values were significantly different between men and women. We evaluated the effect of the interaction between sex and imaging group, by adding it to the logistic regression model. Whenever sex was included in a logistic regression analyses as an explanatory variable, male sex was defined as reference.

All effect sizes are represented by ORs with 95% confidence intervals (CI). The logistic regressions were done with the help of the statsmodels package version 0.10.1 for Python version 3.6.7. Two-sided *p*-values below 0.05 were considered to indicate statistical significance. Reproducibility of ratings was evaluated with percentage of agreement and weighted Cohen’s kappa [41] with help of Stata statistical software [42].

## Results

### Study population

A total of 410 patients were included in the study (Fig. 1). The mean (SD) age was 73.6 (±11) years, 182 (44%) were female, the mean (SD) NIHSS at admission was 4.1 (±5). The population characteristics and cerebrovascular risk factors are summarized in Table 1.

**Table 1:** Baseline characteristics of the included patients

	N	Overall *N* = 410	Female *N* = 182	Male *N* = 228	*p*-value
Age at stroke, years (mean (±SD))	410	73.6 (±11)	75.0 (±12)	72.5 (±11)	0.003
Living alone (N(%))	410	152 (37.0)	88 (48.0)	64 (28.0)	0.05
Education (mean years(±SD))	410	12.1 (±4)	11.4 (±3)	12.7 (±4)	0.0001
NIHSS at admission (0-42) (mean (±SD))	405	4.1 (±5)^a^	4.3 (±5)	3.9 (±5)	0.34
Hemorrhagic stroke (N(%))	395	27 (7.0)	12 (7.0)	15 (7.0)	0.56
Pre-stroke GDS (1-7) (mean (±SD))	407	1.5 (±1)	1.6 (±1)	1.5 (±1)	0.11
Pre-stroke mRS (0-6) (mean (±SD))	408	2.2 (±1)	2.4 (±1)	2.1 (±1)	0.005
TOAST classification (N)	368	368	166	202	0.282^*^
Large vessel disease		39 (10.6)	14 (8.3)	25 (12.4)	
Cardioembolic disease		84 (22.8)	33 (19.9)	51 (25.3)	
Small vessel disease		89 (24.2)	39 (23.5)	50 (24.8)	
Other etiology		7 (1.9)	4 (2.4)	3 (1.5)	
Undetermined etiology		149 (40.5)	76 (45.8)	73 (36.1)	
History of:					
Atrial fibrillation (N(%))	407	65 (16.0)	25 (14.0)	40 (18.0)	0.06
Diabetes (N(%))	407	77 (19.0)	34 (19.0)	43 (19.0)	0.31
Hypertension (N(%))	410	196 (48.0)	82 (45.0)	114 (50.0)	0.02
Hypercholesterolemia (N(%))	407	155 (38.1)	61 (33.7)	94 (41.6)	0.103
Previous stroke (N(%))	410	66 (16.0)	22 (12.0)	44 (19.0)	0.01

Values are displayed as means (SD) and frequencies (%) for all patients and grouped by sex. Values are mean (±SD) for continuous variables and N (%) for binary variables. For group comparisons we used we used Mann-Whitney U test for continuous variables and chi-square test for binary variables. *NIHSS* National Institutes of Health stroke scale, *mRS* modified Rankin Scale, *GDS* Global Deterioration Scale, *SD* standard deviation. Previous stroke is clinically acknowledged stroke. ^a^The median (IQR) of the NIHSS was 3 (0 to 24). *p-value of chi-squared test of TOAST classification contingency table

Comparison of included and excluded patients showed that those included were significantly more likely to have hypercholesterolemia and less likely to have suffered a hemorrhagic stroke (see Supplementary Table 2). Apart from that, the included patients were overall representative for the rest of the Nor-COAST population, and there was no difference in pre-stroke GDS. More details can be found in Supplementary Table 2.

### Imaging findings

Out of all participants, 68% had at least one pathological imaging marker. MTA was present in 30%, WMH in 38%, and lacunes in 35%. The mean age (SD) of the patients with a normal brain was 70 (± 12.9) years compared to 75 (± 10.2) years for those with pathological findings (*p* < 0.001). Table 2 summarizes the imaging findings for the population overall and grouped by sex.

**Table 2:** Pathological MRI findings

	Total n/N (%)	Female n/N (%)	Male n/N (%)	Unadjusted OR (†) (95% CI)	***p***-value	Adjusted OR (††) (95% CI)	***p***-value
**≥1 pathological imaging result**	278/410 (68)	108/182 (59)	170/228 (75)	0.5 (0.33-0.76)	0.001^*^	0.43 (0.28-0.66)	0.0002*
**Pathological WMH**	154/410 (38)	74/182 (41)	80/228 (35)	1.3 (0.9-1.9)	0.25	1.13 (0.7- 1.7)	0.57
**Old infarct**	78/396 (20)	28/175 (16)	50/221 (23)	0.65 (0.4-1.1)	0.10	0.62 (0.4-1.0)	0.07
**Lacunes**	142/410 (35)	51/182 (28)	91/228 (40)	0.6 (0.4-0.9)	0.01^*^	0.53 (0.3-0.8)	0.003*
**Microbleeds**	76/410 (19)	33/182 (18)	43/228 (19)	0.95 (0.6-1.6)	0.85	0.93 (0.6-1.5)	0.78
**Medial temporal lobe atrophy**	125/410 (30)	34/182 (19)	91/228 (40)	0.35 (0.2-0.6)	0.001^*^	0.35 (0.2-0.6)	0.001*
**Posterior atrophy**	44/410 (11)	17/182 (9)	27/228 (12)	0.77 (0.4-1.5)	0.42	0.57 (0.3-1.1)	0.11
**Evans index**	17/410 (4)	6/182 (3)	11/228 (5)	0.67 (0.2-1.9)	0.44	0.7 (0.3-1.9)	0.49

The relationship between sex and pathological imaging findings was modelled using logistic regression with male sex as the reference group. Values are displayed as frequencies (%) for all patients and grouped by sex

(*) indicates significant difference in pathological findings. *WMH* white matter hyperintensities, *path* pathology, *Evans index* measurement of ventricle size, *OR* odds ratio. The analyses were adjusted for age to account for age as a potential confounding variable. (†)Unadjusted OR = OR for women compared to men. (††)Adjusted OR = OR for women compared to men and adjusted for age. * indicating statistically significant odds ratio

### Cognitive assessment

We found that 100 of the 276 patients (36%) with baseline pathological findings also had PCI, whereas 25 of the 143 patients (18.9%) with normal MRI had PCI. The highest percentages of patients with PCI were found in the cerebrovascular group (37.5%) and in the mixed group (44%) while the lowest prevalence of PCI was found in the neurodegeneration group (19%).

The odds ratio (OR) of having PCI for patients with clinically acknowledged previous infarct was 2.9 (95% CI 1.7–5.0, *p* < 0.001), atrial fibrillation was 1.1 (0.6–1.9, *p* = 0.8), silent infarcts was 0.9 (0.5 to 1.8, *p* = 0.8), and hypertension was 1.3 (0.87–2.0, *p* = 0.2).

The OR (95% CI) of PCI for the cerebrovascular groups was 2.5 (1.4–4.5, *p* = 0.001) compared to the normal group. The OR (95%) of PCI was 3.4 (1.9–6.1, *p* = 0.001) for the mixed pathology group and 1.01 (0.5–2.2, *p* = 0.973) for the neurodegeneration group (Table 3).

**Table 3:** Distribution of imaging pathology and pre-stroke cognitive impairment

Pathological Imaging group	N (%)	GDS 1 N(%) 282 (69)	GDS ≥ 2 N(%) 125 (31)	Unadjusted Odds ratio (CI) (†)	***p***-value	Adjusted Odds ratio (CI) (††)	***p***-value
**Normal**	131 (32)	106 (81)	25 (19)	1		1	
**Cerebrovascular pathology**	120 (29)	75 (62.5)	45 (37.5)	2.5 (1.4 to 4.5)	0.001*	2.0 (1.0 to3.7)	0.001*
**Mixed pathology**	99 (24)	55 (56)	44 (44)	3.4 (1.9 to 6.1)	0.001*	2.9 (1.5 to 5.7)	0.001*
**Neurodegeneration**	57 (14)	46 (81)	11 (19)	1.01 (0.5 to 2.2)	0.973	1.2 (0.6 to 2.7)	0.736

*GDS* Global Deterioration Scale, *CI* confidence interval. Values are displayed as absolute frequencies (%) for normal and pathological GDS grouped by dominating imaging pattern (neurodegeneration, vascular pathology, mixed pathology or normal). Counts and frequencies are displayed for each sex separately. (†) Unadjusted odds ratio (CI) for having a pathological GDS compared to the “normal” imaging group. (††) Odds ratio adjusted for age and education

* indicating statistically significant odds ratio. Baseline GDS scores were available for 407 patients out of 410 patients in this study

### Sex differences

Female patients were older (by 2.5 years on average) than men, had less education, and had a higher pre-stroke modified Rankin Scale (mRS) score than men. Women were less likely to suffer from hypertension and had fewer previous clinical strokes (Table 1).

Female patients were less likely to show more than one pathological imaging finding and had fewer lacunes and less pathological MTA (Table 2).

The OR (95% CI) of PCI for women compared to men was 1.2 (0.8 to 1.9, *p* = 0.3), which showed that female sex was not significantly associated with PCI. There was no significant interaction of sex and pathological imaging group. The OR (95% CI) for the interaction of sex and cerebrovascular group was 2.1 (0.65 to 6.5, *p* = 0.217), the OR (95% CI) for the interaction of sex and mixed group was 2.0 (0.59 to 6.8, *p* = 0.257), and the interaction of sex and neurodegenerative group was 1.4 (0.3 to 8.1, *p* = 0.681).

## Discussion

The first aim of this study was to measure pre-stroke neurodegenerative and vascular disease pathology found on brain MRI. Pre-stroke pathology was present in the brain MRIs of more than two thirds of all of the patients in the current study. More than half of all of the patients had markers of small vessel disease exceeding what is normal for their age according to published pathological cut-offs. One in three had pathological neurodegenerative changes. WMH was the most prevalent imaging marker of small vessel disease, and MTA was the most prevalent imaging marker of neurodegeneration. Our results are consistent with previous studies using visual evaluation of similar populations. Imaging markers of both small vessel disease and neurodegeneration are frequently present in patients with stroke [43–45]. Different studies have found MTA in the range of 10–75% of all cases within 14 days to 6 months after stroke [2, 44]. We found MTA in 30% of participants.

Our findings of a 19% prevalence of microbleeds amongst the participants is backed up by other findings generally showing about twice as high a prevalence in studies of cerebrovascular disease (22%) [43] compared to a healthy ageing population (11%) [46].

In studies of patients aged 90 years and older, cerebrovascular disease is the most prevalent non-Alzheimer age-associated pathology [47]. We found that 38% of the study participants had pathological WMH levels. Another prospective stroke study found a comparable prevalence of severe WMH in 30% of participants [36]. In the Rotterdam Scan Study, WMH were present in 95% of the subjects that were included from the general population [48]. It seems there is an overlap between normal aging and small vessel disease in the Rotterdam study. Because of the omnipresence of WMH, we believe that cut-offs should be used whenever possible for interpreting the visual rating scales [23] and they may serve as practical tools in the follow-up of patients’ risk of cognitive impairment [23].

The second aim of our study describe the association between pre-stroke neuroimaging features and PCI. Previous studies have looked into the association of single risk factors or single brain markers and post-stroke dementia, even though it is known that mixed pathologies of neurodegenerative and cerebrovascular changes are common [49]. The highest numbers of patients with PCI were found in the cerebrovascular- and the mixed group that were significantly associated with PCI. Patients with only markers of neurodegeneration were not associated with PCI. This is in line with a study focusing on risk factors for developing dementia that showed that isolated pathologies improve the odds of being resilient [11]. Further might the neurodegeneration be caused by other mechanisms than AD like other neurodegenerative diseases or systemic cardiovascular disease [50]. Robinson et al. also found that in the 90+ Study, resilient patients had less cerebrovascular disease [47]. However, this is in conflict with the hypothesis that most of the cognitive decline in a stroke population is only caused by neurodegenerative changes [51]. The neurodegenerative component in the mixed group has been linked to small vessel disease and not to primary neurodegenerative disease [51, 52], however, a recent study did not confirm this link [53]. This highlights the importance of considering mixed pathology in the etiology of PCI.

PCI was found in 19% of the patients with a normal-appearing MRI and in almost double as many (36%) with a pathological MRI.

There might be different explanations for why only around one-third of the patients with imaging pathology had PCI. First, it might be that the GDS scale has led to a false-negative classification of some patients with PCI. Secondly, this could be explained by differences in brain resilience. Brain resilience relies both on factors affecting cognitive reserve and brain reserve [11]. Previous studies have shown that persons with high brain reserve can tolerate more brain pathology and be cognitively normal for a longer time despite pathological imaging findings on MR. [54]

In the same way, the 19% of the patients with a normal appearing brain may have PCI due to reduced brain resilience. Based on our results, we cannot rule out reduced brain reserve as a consequence of a low total brain volume or impaired structural connectivity [55]. More advanced imaging methods, like diffusion tensor imaging (DTI), may be required to fully understand the relation between brain changes and PCI. Psychological stress has also been shown to impact cognitive outcome after stroke, and to precede cardiovascular disease [56].

The third aim of our study was to investigate possible sex differences in the risk of PCI. Comparing the risk factors for stroke, the women in our study were significantly older than the men, had higher pre-stroke disability (measured by the mRS) and lower education, and more often lived alone. Higher age is consistent with previous stroke studies [16, 19]. However, the women were equally likely as the men to have atrial fibrillation and less likely to have hypertension even though previous studies have shown that women are more frequently affected by both conditions [57]. In this study, more men had hypertension and previous stroke, but not diabetes. This diverges from previous studies that have shown more diabetes in men [57, 58]. The fact that the observed sex-specific risk factor profile in our study differs from previous research might explain why we didn’t observe a higher PCI risk for women. The risk of PCI for women was not increased by the fact that more women lived alone and had less education. These female disadvantages may have been counterbalanced by their lower burden of pathological imaging findings. In fact, women had fewer lacunes and less medial temporal lobe atrophy.

Both men and women in the cerebrovascular and mixed groups had increased ORs for PCI. However, we did not find a significant interaction between sex and imaging pathology group. Our study therefore does not confirm an increased risk of PCI in women as was shown for pre-stroke dementia in the study by Pendlebury et al. [3].

A strength of this study is the large number of prospectively included participants with good quality brain MRIs. The application of cut-off values for visual ratings is another strength that enabled us to categorize visual scores as pathological and differentiate them from normal aging. A further strength of this study is the use of only visual rating scales. Visual rating scales have the advantage of robustness against reduced image quality, they are fast and easy to perform and they do not require any specific software or hardware. This makes them applicable in clinical practice.

A weakness is the lack of cognitive testing on all participants who had MRI scans, which would have given the study more statistical power. It was, however, difficult to recruit some of the sickest patients, also potentially leading to an underestimation of both chronic pathology and PCI. Our patients suffered mainly mild strokes. However, the patient population in the present study has been found to be comparable to that of a general stroke population [59]. This study raises awareness of the high prevalence of brain imaging pathology even in a stroke population suffering mainly minor strokes, which is a strength. Future studies should investigate the association of imaging markers and brain resilience more closely.

## Conclusions

Chronic brain pathology is common in stroke patients. Vascular pathology and mixed pathology are more important than neurodegeneration in the pathogenesis of PCI. There is no significant sex difference for the risk of PCI.

## Supplementary Information

**Additional file 1.**

**Additional file 2.**

**Additional file 3.**

**Additional file 4.**

## Data Availability

The datasets presented in this article are not readily available because of Norwegian regulations and conditions for informed consent. Requests to access the dataset should be directed to IS, ingvild.saltvedt@ntnu.no

## Abbreviations

PCI: Pre-stroke cognitive impairment
GDS: Global Deterioration Scale
NIHSS: National Institute of Health Stroke Scale
MTA: Medial temporal lobe atrophy
WMH: White matter hyperintensities
DWI: Diffusion weighted imaging
FLAIR: Fluid attenuated inversion recovery
STRIVE: Standards for Reporting Vascular Changes on Neuroimaging
PA: Posterior atrophy
EI: Evans’ index
Nor-COAST: Norwegian Cognitive Impairment After Stroke Study
AF: Arterial fibrillation
HT: Hypertension
mRS: Modified Rankin Score
