# Supplementary material for: Pre-stroke cognitive impairment is associated with vascular imaging pathology: a prospective observational study
Source: BMC Geriatr. 2021 Jun 14;21:362. doi: 10.1186/s12877-021-02327-2 (PMC8201706; doi:10.1186/s12877-021-02327-2)
Supplement: Supplementary file 1 — Additional file 1. [file 12877_2021_2327_MOESM1_ESM.docx]

**Supplementary table 1**

| **Hospital** | **Sequence** | **Repetition Time** | **EchoTime** | **InversionTime** | **FA** | **Rows** | **Columns** | **FOV** | **ST** |
| --- | --- | --- | --- | --- | --- | --- | --- | --- | --- |
| Ullevål | T1_BRAVO_iso | 8.2 | 3.2 | 450 | 12 | 256 | 256 | 256x256 | 1 |
|  | CUBE_FLAIR | 8000 | 125.9 | 2092 | 90 | 256 | 256 | 256x256 | 1.2 |
|  | T2-PROPELLER | 6519 | 100.9 | NA | 142 | 512 | 512 | 512x512 | 4 |
|  | SWAN_3D | 37.2 | 23.2 | NA | 10 | 512 | 512 | 512x512 | 2 |
|  | DWI_TETRA | 3500 | 61.2 | NA | 90 | 256 | 256 | 256x256 | 6 |
| Trondheim | T1_MPRAGE_iso | 2300 | 2 | 900 | 9 | 256 | 256 | 256x256 | 1 |
|  | 3D_FLAIR | 5000 | 388 | 1800 | 120 | 256 | 256 | 256x256 | 1 |
|  | T2_TSE | 4200 | 81 | NA | 159 | 448 | 448 | 256x256 | 3 |
|  | SWI | 29 | 30 | NA | 15 | 512 | 384 | 512x384 | 2 |
|  | DWI | 6400 | 76 | NA | 90 | 128 | 128 | 128x128 | 4 |
| Haukeland | T1_MPRAGE_iso | 2300 | 2 | 900 | 9 | 256 | 256 | 256x256 | 1 |
|  | 3D_FLAIR | 5000 | 386 | 1800 | 120 | 256 | 256 | 256x256 | 1 |
|  | T2 | 4370 | 73 | NA | 150 | 448 | 448 | 448x448 | 3 |
|  | SWI | 29 | 20 | NA | 15 | 512 | 384 | 512x384 | 2 |
|  | DWI | 6400 | 76 | NA | 90 | 130 | 130 | 130x130 | 4 |
| Vestre Viken | T1-3D | 7.5 | 3.4 | NA | 8 | 320 | 320 | 320x320 | 1.1 |
|  | 3D_FLAIR | 4800 | 284.1 | 1660 | 90 | 288 | 288 | 288x288 | 1.1 |
|  | T2 | 7069 | 100 | NA | 90 | 560 | 560 | 560x560 | 4 |
|  | SWI | 51 | 0 | NA | 20 | 672 | 672 | 672x672 | 2 |
|  | DWI | 4773 | 101 | NA | 90 | 224 | 224 | 224x224 | 4 |
| Ålesund | T1_3D | 25 | 4.6 | NA | 30 | 320 | 320 | 320x320 | 1 |
|  | 3D_FLAIR | 4800 | 250.5 | 1660 | 90 | 432 | 432 | 432x432 | 1.3 |
|  | T2 | 5463 | 100 | NA | 90 | 560 | 560 | 560x560 | 5 |
|  | VEN_BOLD | 24.6 | 34.7 | NA | 10 | 512 | 512 | 512x512 | 1 |
|  | DWI | 4623.9 | 108.3 | NA | 90 | 176 | 176 | 176x176 | 4 |

FA = flip angle. ST= slice thickness. FOV= field of view. DWI = diffusion weighted imaging. FLAIR= fluid attenuated inversion recovery. 3D = three dimensional. SWI/ SWAN/VEN BOLD = susceptibility weighted imaging. TSE= turbo spin echo
