# Supplementary material for: Pre-stroke cognitive impairment is associated with vascular imaging pathology: a prospective observational study
Source: BMC Geriatr. 2021 Jun 14;21:362. doi: 10.1186/s12877-021-02327-2 (PMC8201706; doi:10.1186/s12877-021-02327-2)
Supplement: Supplementary file 2 — Additional file 2. [file 12877_2021_2327_MOESM2_ESM.docx]

|  | Overall |  |  | Female |  |  | Male |  |  |
| --- | --- | --- | --- | --- | --- | --- | --- | --- | --- |
|  | Study MRI  N=410 | No  Study MRI  N=405 | p-value | Study MRI  N=182 | No  Study MRI  N=183 | p-value | Study MRI  N=228 | No  Study MRI  N=222 | p-value |
| Age at stroke, years (mean (±SD)) | 73.1 | 73.9 | 0.37 | 74.5 | 76.9 | **0.06** | 72.0 | 71.4 | 0.57 |
| Living alone (N (%)) | 152 (37.1) | 146 (36.1) | 0.58 | 88 (48.4) | 105 (57.4) | 0.18 | **64 (28.1)** | 41 (18.5) | **0.04** |
| Education (mean years(±SD)) | 12.1 (3.7) | 11.8 (3.8) | 0.29 | 11.4 (3.4) | 11.1 (3.5) | 0.43 | 12.7 (3.8) | 12.4 (3.9) | 0.49 |
| NIHSS at admission (0-42) (mean (±SD)) | 4 (4.9) | 5 (6.9) | **0.02** | 4.2 (4.9) | 5.6 (7.3) | **0.03** | 3.8 (4.9) | 4.4 (6.4) | 0.29 |
| Hemorrhagic stroke (N (%)) | 29 (7.1) | 53 (13.1) | **0.02** | 12 (6.6) | **30 (16.4**) | **0.01** | 17 (7.5) | 23 (10.4) | 0.56 |
| Pre- stroke GDS (1-7) (mean (±SD)) | 1.5 (0.9)  N=407 | 1.7 (1.1)  N=396 | 0.10 | 1.6 (1.1)  N=181 | 1.8 (1.2)  N=181 | 0.22 | 1.5 (0.9)  N=226 | 1.6 (1.1)  N=215 | 0.29 |
| Pre-stroke mRS (0-6) (mean (±SD)) | 0.9 (1.1)  N=410 | 1.07 (1.2)  N=401 | **0.08** | 1.1 (1.2) | 1.3 (1.3)  N=181 | 0.12 | 0.8 (1.0)  N=228 | 0.9 (1.2)  N=220 | 0.34 |
| Atrial fibrillation (N (%)) | 64 (15.6) | 78 (19.3) | 0.14 | 25 (13.7) | 33 (18.0) | 0.26 | 39 (17.1) | 45 (20.3) | 0.24 |
| Diabetes (N(%)) | 77 (18.8) | 59 (14.6) | **0.09** | 34 (16.7) | 16 (8.7) | **0.01** | 43 (18.9) | 43 (19.4) | 0.68 |
| Hypertension (N(%)) | 196 (47.8) | 206 (50.9) | 0.232 | 82 (45.1) | 95 (51.9) | 0.19 | 114 (50) | 111 (50) | 0.36 |
| Hypercholesterolemia (N(%)) | **155 (37.8)** | 109 (26.9) | **0.003** | 61 (33.5) | 45 (24.6) | 0.12 | 94 (41.2) | 64 (28.8) | **0.02** |

**Supplementary table 2**
