# Supplementary material for: Pre-stroke cognitive impairment is associated with vascular imaging pathology: a prospective observational study
Source: BMC Geriatr. 2021 Jun 14;21:362. doi: 10.1186/s12877-021-02327-2 (PMC8201706; doi:10.1186/s12877-021-02327-2)
Supplement: Supplementary file 3 — Additional file 3. [file 12877_2021_2327_MOESM3_ESM.docx]

**Supplementary table 3**

**Results of reliability testing**

The following table compares the results of the Nor-COAST reliability assessment with previously published results.

| **Inter-rater**  **agreement** | **Fazekas** | **MTA** | **PA** | **Microbleeds** | **Evans** | **Lacunes** |
| --- | --- | --- | --- | --- | --- | --- |
| Percent agreement (%) | 94.83 | 88.83 | 87.36 | 96.15 | NA | 72.41 |
| Weighted Kappa – Nor-COAST  % (Std. error) | 0.88  (0.14) | 0.55  (0.10) | 0.34  (0.10) | 0.88  (0.19) | 0.95 (ICC)  (0.90 to 0.98) | 0.29  (0.16) |
| Reliability^*^ - Literature | 0.70^(1)^ | 0.72 to 0.84^(2)^ | 0.65 to 0.84^(2)^ | 0.44  (0.32 to 0.56) | 0.91^(3)^ | NA |
| **Intra-rater**  **agreement** | **Fazekas** | **MTA** | **PA** | **Microbleeds** | **Evans** | **Lacunes** |
| Percent agreement (%) | 90.00 | 91.3 | 88.33 | 77.78 | NA | 86.67 |
| Weighted Kappa – Nor-COAST  % (Std. error) | 0.77  (0.14) | 0.66  (0.13) | 0.29  (0.13) | 0.15  (0.16) | 0.96 (ICC)  (0.93 to 0.98) | 0.42  (0.18) |
| Reliability^*^ - Literature | 0.79^(1)^ | 0.83 to 0.91^(2)^ | 0.93 to 0.95^(2)^ | 0.72^(4)^  (0.62 to 0.82) | >0.9(ICC)^(5)^ | NA |

^* Reliability is estimated with weighted kappa when not stated otherwise.^

1. Gouw AA, van der Flier WM, Fazekas F, van Straaten EC, Pantoni L, Poggesi A, et al. Progression of white matter hyperintensities and incidence of new lacunes over a 3-year period: the Leukoaraiosis and Disability study. Stroke. 2008;39(5):1414-20.

2. Harper L, Barkhof F, Fox NC, Schott JM. Using visual rating to diagnose dementia: a critical evaluation of MRI atrophy scales. Journal of neurology, neurosurgery, and psychiatry. 2015;86(11):1225-33.

3. Reinard K, Basheer A, Phillips S, Snyder A, Agarwal A, Jafari-Khouzani K, et al. Simple and reproducible linear measurements to determine ventricular enlargement in adults. Surgical neurology international. 2015;6:59.

4. Gregoire SM, Chaudhary UJ, Brown MM, Yousry TA, Kallis C, Jäger HR, et al. The Microbleed Anatomical Rating Scale (MARS): reliability of a tool to map brain microbleeds. Neurology. 2009;73(21):1759-66.

5. Brix MK, Westman E, Simmons A, Ringstad GA, Eide PK, Wagner-Larsen K, et al. The Evans' Index revisited: New cut-off levels for use in radiological assessment of ventricular enlargement in the elderly. European journal of radiology. 2017;95:28-32.
