# Supplementary material for: Pre-stroke cognitive impairment is associated with vascular imaging pathology: a prospective observational study
Source: BMC Geriatr. 2021 Jun 14;21:362. doi: 10.1186/s12877-021-02327-2 (PMC8201706; doi:10.1186/s12877-021-02327-2)
Supplement: Supplementary file 4 — Additional file 4. [file 12877_2021_2327_MOESM4_ESM.docx]

**Supplementary figure 1**

**Overview of the reliability testing process**


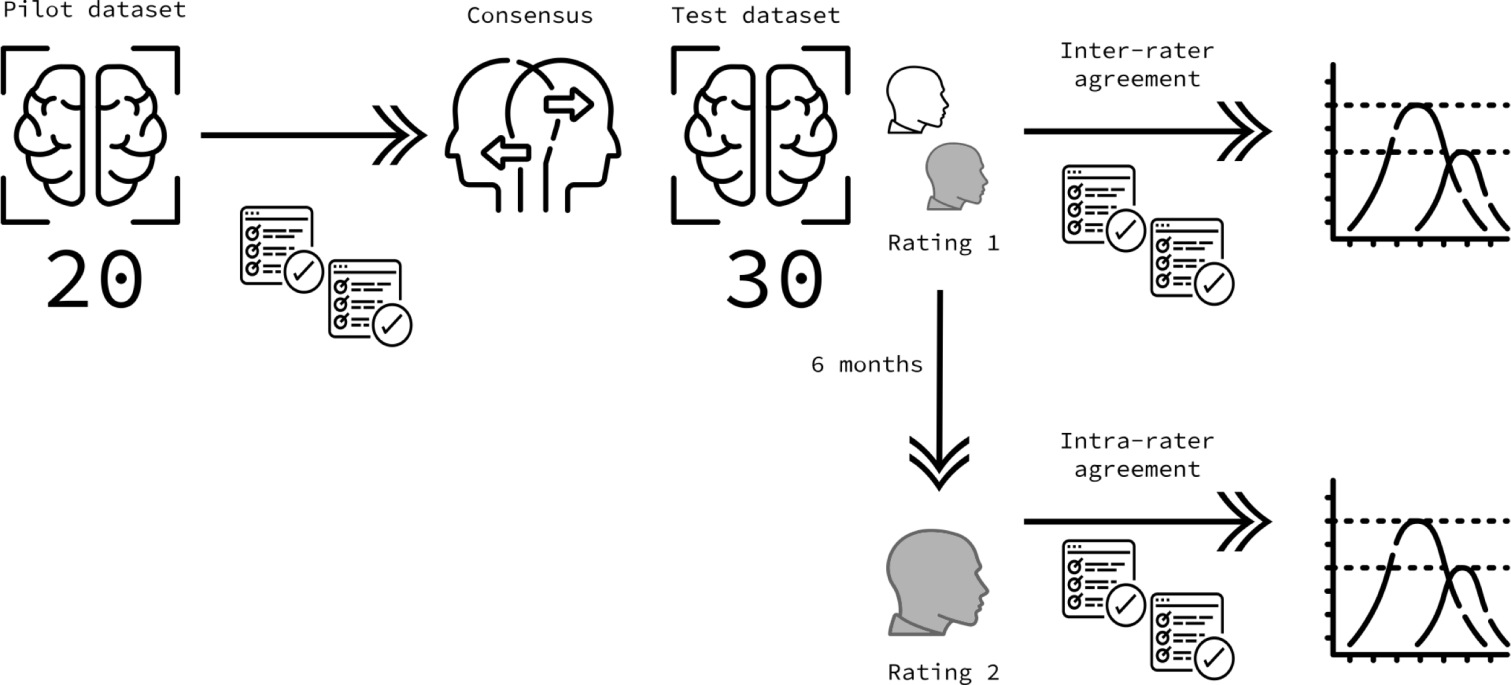


Supplementary figure 1: Overview of the reliability testing process.
